# Holmium laser-assisted laparoscopic partial cystectomy for bladder cancer: a single-institutional pilot study with technical feasibility and short-term oncological outcome

**DOI:** 10.1186/s12885-022-09308-7

**Published:** 2022-02-21

**Authors:** Kang Sup Kim, Sang Hoon Kim, Hyuk Jin Cho, Hong Jin Sur, Yong Sun Choi

**Affiliations:** 1 Department of Urology, Incheon St. Mary’s Hospital, College of Medicine, The Catholic University of Korea, Incheon, Korea; 2 Department of Urology, Eunpyeong St. Mary’s Hospital, College of Medicine, The Catholic University of Korea, 1021, Tongil-ro, Eunpyeong-gu, Seoul, 03312 Korea; 3 Department of Urology, Seoul St. Mary’s Hospital, College of Medicine, The Catholic University of Korea, Seoul, Korea

**Keywords:** Muscle-invasive bladder carcinoma, Partial cystectomy, Holmium laser

## Abstract

**Background:**

In selected patients with bladder cancer, partial cystectomy is an alternative treatment for bladder preservation with fair oncologic result. During partial cystectomy, tumor margin demarcation is difficult. Various methods were adopted, however, there is no standard for tumor margin demarcation. We aimed to introduce and provide our experience with holmium laser-assisted method with ten patients.

**Methods:**

From March 2016 and February 2019, patients who want partial cystectomy for bladder cancer were enrolled in this study. Inclusion criteria were stage T2 or T3 disease and tumor location restricted within the dome, and lateral, posterior side of the bladder were included. Transurethral holmium laser-assisted mucosal incision was made and deepened until perivesical fat. Minimal Safety margin for 5-10 mm were spared, and tumor removal was done laparoscopically.

**Results:**

Ten patients underwent holmium laser-assisted laparoscopic partial cystectomy. All procedures were done without complication. The tumor locations were laterally in seven patients, dome in two patients, and posterior wall in one patient. Pathologic examination of surgical margin showed no cancer cell involvement in all cases. There were no recurrences or metastases for 12 months follow up.

**Conclusions:**

Holmium laser-assisted laparoscopic partial cystectomy is effective and safe technique in carefully selected patients. To achieve precise and appropriate surgical margin during the laparoscopic partial cystectomy, holmium laser resection provides feasible and safe method that assists in bladder incision with minimal ureteral orifice involvement.

**Trial registration:**

Retrospectively registered.

## Background

Radical cystectomy is the treatment of choice in advanced bladder cancer, but the surgeon and the patient may hesitate because of the high complication rates, high surgical risks, and lower postoperative quality of life [1–4]. In selected patients with bladder cancer, partial cystectomy can obtain similar oncologic results with lower morbidity and better quality of life than radical surgery [5]. During the partial cystectomy, precise incision line decision, far distant from the tumor, requires either palpation of mass or a “smart guess” based on previous knowledge of tumor location. But, in many cases, most of the tumor has been removed by previous transurethral resection, making it impossible to palpate the tumor. Furthermore, with bladder out obstruction condition, the bladder wall may be often thickened, making exact tumor palpation difficult. Alternatively, contralateral or midline incision can be considered, and the tumor or previous transurethral resection scar might be identified from inside of bladder. This procedure can compromise the surgical margins or even go through the tumor itself and two suture lines may require for closure [6].

.We now report our experience using the holmium laser-assisted laparoscopic partial cystectomy in treating patients with locally advanced bladder cancer. This procedure is a simple, requires one incision, and provide maximal surgical margin even the mass is near the ureteral orifice.

## Methods

### Ethics statement

This study was reviewed and approved by the Institutional Review Board and Medical Ethics Committee of the Catholic University of Korea (approval number: OC21RASI0074). The requirement for informed consent was waived because of the retrospective nature of this study, and this study was conducted in accordance of Declaration of Helsinki.

### Patient selection

This pilot study was conducted between March 2016 and February 2019. The inclusion criteria were age 18 to 75 years, ability to understand the details of the written informed consent. The patients who checked cystoscopic examination to confirm the bladder tumor and exact location of tumor site were enrolled. After the confirmation of tumor and tumor site, patients received abdomen-pelvis enhance CT for lymph node enlargement, any abdomen metastasis, and depth of invasion evaluation. Chest CT scan was done simultaneously before surgery, to find out the lung metastasis. This surgery can be performed only in carefully selected of patients. We selected the patients so carefully to avoid unintended result. As a result, all histologic type of bladder cancer is typical transitional cell carcinoma. There was no variant histology, multifocality, and LN enlargement. Patients with one or two solitary tumors, no metastasis, stage T2 or T3 disease and tumor location restricted within the dome, and lateral, posterior side of the bladder were included. The tumor inside of trigone or base of the bladder, and tumor with ureteral invasion were excluded because of the partial cystectomy inability. Furthermore, the depth of invasion should not be T4 in preoperative CT scan, and the patient refusal of radical cystectomy for any reason.

### Study endpoints

The primary endpoint of this study was to evaluate the efficacy of Holmium laser-assisted laparoscopic partial cystectomy as measured by the safety margin status of tumor specimen and 12 months short term follow up. The secondary endpoint was the safety of Holmium laser-assisted laparoscopic partial cystectomy assessed by hemoglobin change during surgery, operation time, and postoperative complications.

### Surgical intervention

Two experienced urologists performed both Holmium laser-assisted cystoscopic incision, and transperitoneal partial cystectomy, simultaneously. Antibiotics were administered prophylactically to all patients.

Under general anesthesia, the patient laid in lithotomy position. The transperitoneal approach with three ports was applied for bladder mobilization and standard pelvic lymph node dissection by one urologist. During the transperitoneal approach, the other surgeon inserted cystoscope into bladder. After the identification of the tumor, the bladder was over-distended with saline and we can visualize and decide enough surgical incision line precisely, and safely. During the cystoscope surgery, the bladder was over-distended to achieve adequate surgical margin from the mass lesion. Over-distended bladder makes detrusor muscle thin and gives the operator to gain more bladder capacity, more distance from the mass lesion. The Holmium laser mucosal incision was made by surrounding the tumor with 0.5 to 1.0 cm of normal bladder mucosal safety distance. The after the mucosal incision, the incision was deepened until the perivesical fat layer and peritoneal covering. Perivesical layer. With this technique, we can gain enough safe surgical margins while maintaining bladder capacity. Reaching the fat layer, the operative lights are then turned off, and we made the operation room dark. The laparoscopic surgeon then aimed the laparoscopic scope to peri-vesical transilluminating red laser circle. Along the pre-laser incised demarcation, the cold-scissor used en-block partial cystectomy was done. The specimen was immediately collected with lap-bag for cancer spillage prevention. Additional specimen is submitted for frozen section examination to confirm the absence of microscopic disease at the margin. The defect was then closed with layer by layer fashion, with 2-0 V-Loc (Covidien) suture water tightly. After bleeding control and urine leakage test, J-P drain was inserted and 18Fr Foley catheter was indwelled.

Seven days after the operation, cystogram was checked to confirm urine leakage. Foley catheter was removed after cystogram, and pathology was checked. Adjuvant therapy was done according to the pathology result. Intravesical Bacillus Calmette-Guerin (BCG) instillation was provided to patients with carcinoma in situ. If the CIS components were written in the patient pathologic report, we start the intravesical BCG after 14 days after the surgery to prevent extravesical leakage or systemic toxicity. The BCG administration schedule is standard 6 weeks induction and maintenance course. We used the OncoTICE® (TICE strain) 12.5 mg as BCG therapy. In patients with pT3 or lymph node positive disease, platinum-based adjuvant systemic chemotherapy was considered with oncologist. Because of our country insurance system, the neoadjuvant and adjuvant chemotherapy were done in internal medicine oncology department. So, unfortunately, we have limited accessibility to those records, and have limitation about neoadjuvant chemotherapy. Patients were checked at every 3-months follow-up schedule including cystoscopy, CT scan, urine cytology.

## Results

Holmium laser-assisted laparoscopic partial cystectomy was successfully performed in 10 patients. No patient required conversion to open surgery. One patient experienced frozen margin cancer positive and additional resection was required during the operation. Additional resection frozen result was cancer negative and the final pathology was negative either. Demographic data and perioperative results are presented in Table 1.

**Table 1:** Demographics and perioperative parameters

Case	Age	Sex	ASA score	Operation time	EBL	Hemoglobin change	Hospital stay
1	57	M	3	190	50	2.1	5
2	63	M	2	140	60	1.8	4
3	62	F	3	180	90	3.2	5
4	66	M	2	130	80	2.5	5
5	54	F	3	180	50	1.8	6
6	49	M	1	120	50	2.1	5
7	72	M	2	190	40	1.4	6
8	69	M	3	190	60	2.1	5
9	71	F	3	150	30	1.2	5
10	70	M	2	170	40	1.5	6

The mean age of the patients was 63.3 (range 49-72) years with seven males and three females. The median American Society of Anesthesiologists physical status classification varies one in one patient, two in four patients, and three in five patients. The mean operation time was 164 (range 120-190) minutes. The mean hemoglobin change between preoperative and postoperative was 1.97 g/dL (range 1.2-3.2 g/dL). The mean hospital stay was 5.2 (range 4-6) days. The postoperative complications were all classified as Modified Clavien Classification grade 2 and controlled with regular antibiotics and analgesics. Blood transfusion was not required in all patents during and postoperative period.

The tumor characteristics were shown in Table 2. Timor size was measured with largest diameter and varies from 14 to 35 mm. Most of the tumors were in lateral position. And there was pathological upstaging in 3 patients. The surgical margin status of safety margin was negative in all patients.

**Table 2:** The tumor characteristics and pathologic feature

Case	Tumor size (mm)	Tumor location	Preoperative stage	Postoperative stage	Tumor grade	Surgical margin cancer involvement
1	22	Rt. lateral	T2	T2	High	Negative
2	24	Lt. lateral	T2	T2	High	Negative
3	32	Dome	T2	T2	High	Negative
4	19	Posterior	T2	T3	High	Negative
5	31	Lt. lateral	T3	T3	High	Negative
6	14	Lt. lateral	T2	T3	High	Negative
7	35	Rt. lateral	T3	T3	High	Negative
8	26	Dome	T2	T2	High	Negative
9	21	Lt. lateral	T2	T3	High	Negative
10	27	Lt. lateral	T3	T3	High	Negative

**Fig. 1:**
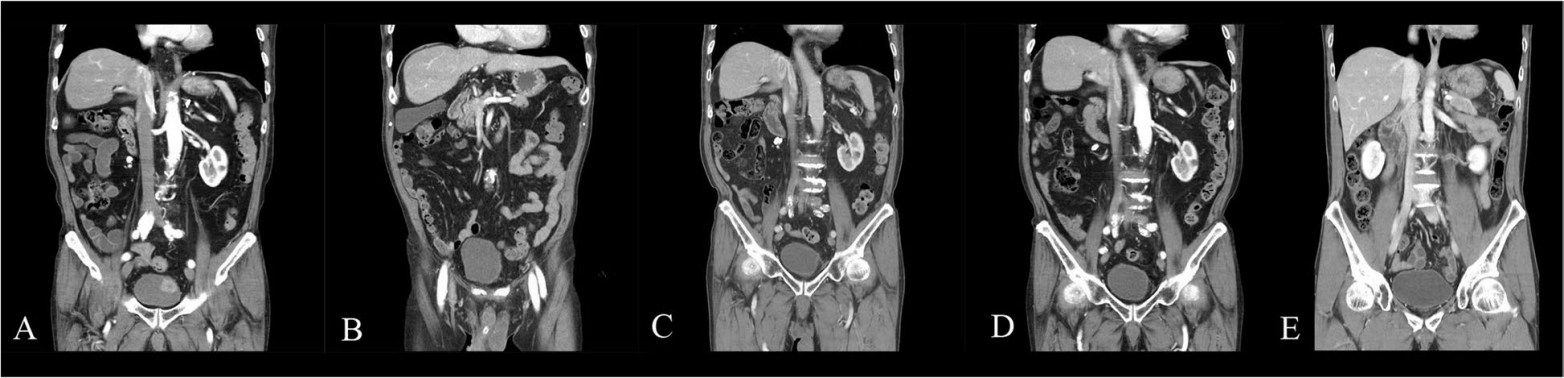
CT scan of partial cystectomy patient. **A** Preoperative CT scan, **B** Postoperative 3 months, **C** Postoperative 6 months, **D** Postoperative 9 months, **E** Postoperative 12 months

**Fig. 2:**
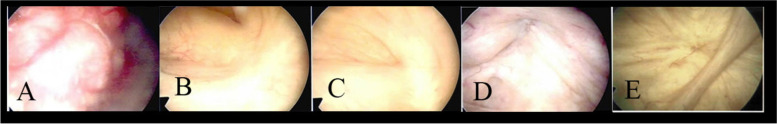
Cystoscope of partial cystectomy patient. **A** Preoperative Cystoscopy, **B** Postoperative 3 months, **C** Postoperative 6 months, **D** Postoperative 9 months, **E** Postoperative 12 months

All patients received adjuvant chemotherapy and tolerable during the follow up period. And, during the 12 months follow up, there were no recurrences or metastases in all patients in their cystoscopy, urine cytology and CT scan (Figs. 1 and 2).

## Discussion

During the last decade, the Food and Drug Administration’s (FDA) approval of laser therapy for patients with recurrent superficial bladder carcinoma (neodymium:YAG [Nd:YAG], 1984; Argon, 1987; and KTP, 1988) has allowed urologist to simplify their treatment of patients with this disease [7]. Despite nowadays’ standard procedure for staging and treating non-muscle invasive bladder tumor by transurethral resection of bladder tumor (TURB) via a wire loop, laser resection technique for bladder tumor came back in focus with the introduction of holmium laser [8, 9]. The holmium laser was first used urologist in the early 1990s as holmium laser enucleation of the prostate (HoLEP) [10, 11]. In a typical endourological setting, the onset of vaporization is in the irrigant next to the fiber tip, where a steam bubble is generated with each laser pulse. Within this setting, the laser induced steam bubbles separated soft tissue layers apart [10]. This tissue effect is rapid, and hemostasis of the holmium laser is excellent.

In standard partial cystectomy, the initial incision line should be decided carefully. The incision line should not be too far or not too close from the bladder tumor. Physical tactile sensation or “smart guess” was the only means of getting information of incision line decision [6]. However, these methods are unable in laparoscopic procedures or has high risk for wrong incision [12]. Various techniques were developed to acquire precise partial cystectomy incision line. Previous study introduced cystoscopic light or flexible cystoscope to confirm exact tumor location during the operation [6]. In another study, preoperative India ink tattooing was applied either [13]. Using India ink, preoperative tumor margin cystoscopic tattooing was performed 1 cm away from the tumor margin, and the tattooing area was easily identified under laparoscopic view. The results of those methods were fair, but those procedures require open conversion or inconvenient for patient. Knoedler et al. executed a matched-control analysis study comparing partial cystectomy to radical cystectomy [14]. Patients undergoing partial cystectomy had a single bladder tumor without associated CIS. Patients were matched based on gender, age, and pathological stage. Patients undergoing partial cystectomy were less likely to have multifocal bladder cancers on final pathology as compared to radical cystectomy (15.1% vs. 32.9%). 38% of patients who underwent a partial cystectomy occurred an intravesical recurrence. 5% of partial cystectomy patients experienced a pelvic recurrence. At a mean follow-up of 6.2 years, 81% of patients maintained an original bladder. Most significant, no differences existed between partial cystectomy and racial cystectomy with regard to 10-year metastasis free survival (61% vs. 66%), cancer-specific survival (CSS) (58% vs. 63%), or overall survival (36% vs. 36%).

In this manuscript, we introduced the new rendezvous techniques with holmium laser en block resection and laparoscopic partial cystectomy. While cystoscopic mucosal laser incision, the bladder is fully filled with saline and tumor was monitored under direct vision. As the resection of bladder progresses, the bladder is more distended, and the resection is getting deepened and easier. The tumor safety margin can be easily acquired with laser resection without any complication such as ureteral orifice injury. Because of the low penetration energy, and simultaneous hemostasis, no obturator nerve reflex or bleeding was observed during the operation. The laser resection margin was clear, showed excellent hemostasis, and offers a better laparoscopic vision. In lateral wall mass near the ureteral orifice, the standard partial cystectomy should be performed with caution to prevent ureteral orifice injury. But in our holmium laser-assisted method, the tumor margin can be spared maximally with ureteral orifice safe.

In partial cystectomy, demarcation of mass is difficult and most important step for operation. We are the first group for using holmium laser in laparoscopic partial cystectomy. Our holmium laser-assisted method provides direct tumor vision, excellent hemostasis, and maximal safety margin without any complication.

The limitations of this study are first, partial cystectomy candidate. The tumor site should be lateral, dome or posterior. Base or ureteral invasion mass cannot be treated with partial cystectomy and need radical surgery. Partial cystectomies are rare operations, especially in teaching centers, and it may take years to recruit a larger series [15, 16]. Second, the small sample size is the main limit of the study. Because this manuscript is a pilot study, further evaluation with large population should be followed. Third, its short follow up period is the limitation of study. Even postoperative and 12 months follow up oncologic results were fair the long term follow up should be needed. Large population with randomized, controlled study should be performed to determine efficacy and safety of holmium laser-assisted laparoscopic partial cystectomy.

## Conclusion

Holmium laser-assisted laparoscopic partial cystectomy is effective and safe technique in carefully selected patients. To achieve precise and appropriate surgical margin during the laparoscopic partial cystectomy, holmium laser resection provides feasible and safe method that assists in bladder incision with minimal ureteral orifice involvement. This is a pilot study, and additional studies with prospective, longer follow up duration are mandatory to confirm standard Holmium laser-assisted laparoscopic partial cystectomy technique.

## Data Availability

The datasets generated and analyzed during the current study are not publicly available due to institutional data protection law and confidentiality of patient data but are available from the corresponding author on reasonable request in person.

## Abbreviations

BCG: Bacillus Calmette-Guerin
TURB: Transurethral resection of bladder tumor
HoLEP: Holmium laser enucleation of the prostate
